# Influence of Multiple Castings and Surface Treatments on Metal-Ceramic Bond Strength and Surface Composition Variations of Nickel-Chromium (Ni-Cr) Alloy

**DOI:** 10.7759/cureus.93481

**Published:** 2025-09-29

**Authors:** Leishangthem Brainy Chanu, Angurbala Dhal, Tapan K Patro, Lokanath Garhnayak, Ullash Kumar, Doyir Tasar

**Affiliations:** 1 Department of Prosthodontics, SCB Dental College and Hospital, Cuttack, IND; 2 Department of Prosthodontics and Crown and Bridge, SCB Dental College and Hospital, Cuttack, IND; 3 Department of Prosthodontics and Implantology, SCB Dental College and Hospital, Cuttack, IND

**Keywords:** metal-ceramic bond, multiple casting, ni-cr alloy, surface composition, surface treatment

## Abstract

Aim

The aim of this study was to investigate the effect of multiple castings of nickel-chromium (Ni-Cr) base metal alloy and surface treatment on the metal-ceramic shear bond strength and surface chemical composition.

Material and methods

A total of 108 specimens (5 × 10 mm) were prepared and divided into six groups (n = 18). Three groups of Ni-Cr (4-all; Ivoclar, Schaan, Liechtenstein) alloy specimens (5 × 8 mm) were cast as follows: 100% fresh alloy (C0), addition of 50% by weight remnant of C0 (C1), and addition of 50% by weight remnant of C1 (C2). Within each alloy group, half of the specimens were treated with oxidative heat treatment only (S0), and the remaining half were treated with oxidative heat treatment combined with air abrasion (S1). Feldspathic ceramic (IPS Classic Ceramic; Ivoclar) was applied (5 × 2 mm), and shear bond strength was evaluated using a universal testing machine. Fracture surfaces were analyzed with a stereomicroscope. One specimen from each group was further evaluated for surface composition using scanning electron microscopy (SEM) coupled with energy dispersive X-ray spectrometry (EDS). Data were analyzed using two-way ANOVA followed by the post hoc Tukey test with Bonferroni’s correction.

Results

Group C0 exhibited the highest mean shear bond strength, followed by C1, which was not significantly different. Both groups had significantly higher values compared to C2 (P < 0.05). Fracture modes were predominantly mixed in C0 and C1, whereas C2 showed a higher frequency of adhesive failures. SEM-EDS analysis revealed a gradual decline in molybdenum (Mo) and Cr content with successive recasting.

Conclusions

Recasting up to one generation with 50% fresh alloy supplementation maintains clinically acceptable metal-ceramic bond strength. Recasting beyond the first generation significantly compromises bond integrity. Group S1 showed higher bond strength than Group S0; however, the difference was not statistically significant, and its advantage remains uncertain.

## Introduction

Manufacturers of dental alloys generally advise against the reuse of previously melted alloy [1], although recasting base metal alloys in dentistry is often practiced to reduce costs [2-5], conserve natural resources, and minimize environmental impact [6,7]. However, the addition of previously used alloy remnants to reduce material waste raises questions about its influence on the integrity of the metal-ceramic interface, a critical determinant of clinical success.

The recasting process can be completed in a single stage by merging sprues and surplus material or in multiple steps by combining recast alloys with fresh alloy in varying ratios [6]. Nonetheless, the reuse of melted alloys remains a subject of debate within dental practice. Impurities from previously melted alloys may affect the properties of recast metal. Some studies have reported no substantial changes in mechanical properties following recasting [8]; however, several others have shown that recasting noble and base metal alloys can significantly alter key properties such as corrosion resistance, color, hardness, yield strength, metal-ceramic bond strength [9], and surface microstructures due to the loss of critical elements during evaporation or oxidation [10].

Surface treatments of various base metal alloys have been investigated to improve the metal-ceramic bond, including oxidative heat treatment, airborne particle abrasion, bonding chemicals, and rotary tools [11-17]. The thickness of the oxide layer formed during oxidation for porcelain firing is a critical factor in the performance of base metal casting alloys. Degradation of this oxide layer can lead to failure of the bond between the base metal and ceramic. Conversely, oxidative heat treatment can eliminate crystal defects, contributing to a stabilized microstructure, reduced residual stress, and enhanced metal-ceramic bond strength [18].

Researchers have developed numerous tests to evaluate metal-ceramic bond strength, classified according to the type of stress applied, including shear, tension, flexure, and torsion tests [19]. Some authors consider the shear test the most reliable method for measuring the bond between two materials [19-21], and it is commonly used to assess the bonding strength between alloys and dental ceramics [9,20,22-24].

Since the effects of oxidative heat treatment and oxidative heat treatment combined with air abrasion on the bond strength of multiple recast alloys have not been studied, the purpose of this study was to assess whether successive recasting of nickel-chromium (Ni-Cr) alloys and variations in surface treatment affect the strength and integrity of metal-ceramic bonding. The null hypothesis of this study was that there would be no difference in metal-ceramic shear bond strength of ceramic to Ni-Cr alloy after multiple castings and various surface treatments.

## Materials and methods

To evaluate the metal-ceramic bond strength of fresh and recast Ni-Cr alloys (composition in wt%: Ni: 61.4, Cr: 25.7, molybdenum (Mo): 11.0, silicon (Si): 1.5, manganese (Mn): <1.0, aluminum (Al): <1.0, and carbon (C): <1.0), a total of 108 specimens were prepared and divided into six groups: C0S0, C0S1, C1S0, C1S1, C2S0, and C2S1 (n = 18).

According to the literature, Ucar et al. [8] reported that the mean ± SD shear bond strength in the cast-once group was 577.8 ± 139.4 MPa, while that in the cast-twice group was 494.8 ± 77.6 MPa. With the null hypothesis H₀: m₁ = m₂ versus the alternative H₁: m₁ = m₂ + d, where d is the difference between two means, and n₁ and n₂ are the sample sizes of Groups I and II (N = n₁ + n₂, with ratio r = n₁/n₂ = 1 for equal group sizes), the required sample size was estimated using the formula:

$N = \frac{(1+r)(Z_{\alpha/2} + Z_{1-\beta})^2 \sigma^2}{r d^2}$

Using α = 0.05 (Zα/2 = 1.96), power = 90% (Z1-β = 1.28), r = 1, σ = 108.5, and d = (577.8 - 494.8), the calculated sample size was:

$N = \frac{(1+1)(1.96+1.28)^2 (108.5)^2}{1 \times (577.8 - 494.8)^2} \approx 35$

For metal alloy casting, wax patterns measuring 5 × 8 mm in diameter were cast using an LC CAST 60A machine (Confident Dental Equipments Pvt Ltd, Bengaluru, India) at a temperature of 1405-1465 °C for group C0. For group C1, 50% of the remnants from C0, including the sprue and sprue former, were combined with 50% fresh alloy. For group C2, 50% of the remnants from C1 were added to 50% fresh alloy. All samples were cleaned in distilled water using an ultrasonic cleaner for 30 minutes at 37 °C and then dried with absorbent paper. Subsequently, the specimens underwent air abrasion with 110 µm aluminum oxide particles at 0.2 MPa for five seconds, maintaining a distance of 2.0 cm and an angle of approximately 45°. After abrasion, samples were ultrasonically cleaned in isopropyl alcohol for 10 minutes.

Based on surface treatment, specimens were further divided into two groups: S0 and S1. For the S0 group, oxidative heat treatment was performed with a heating rate of 55 °C/min until reaching 980 °C, maintaining the peak temperature for five minutes. For the S1 group, specimens underwent the same oxidative heat treatment followed by air abrasion and ultrasonic cleaning in isopropyl alcohol for 10 minutes. Feldspathic ceramic (IPS Classic Ceramic; Ivoclar, Schaan, Liechtenstein) was layered on one end of the metal rod (dimensions 5 × 2 mm) and fired in a ceramic furnace according to the manufacturer’s standard protocol [10]. A uniform ceramic thickness of 2 mm was achieved by finishing the surface with an Arkansas stone. Ceramic glazing was performed by applying a thin, even layer of glaze over the ceramic surface, followed by firing in a furnace at the manufacturer’s recommended temperature.

**Figure 1:**
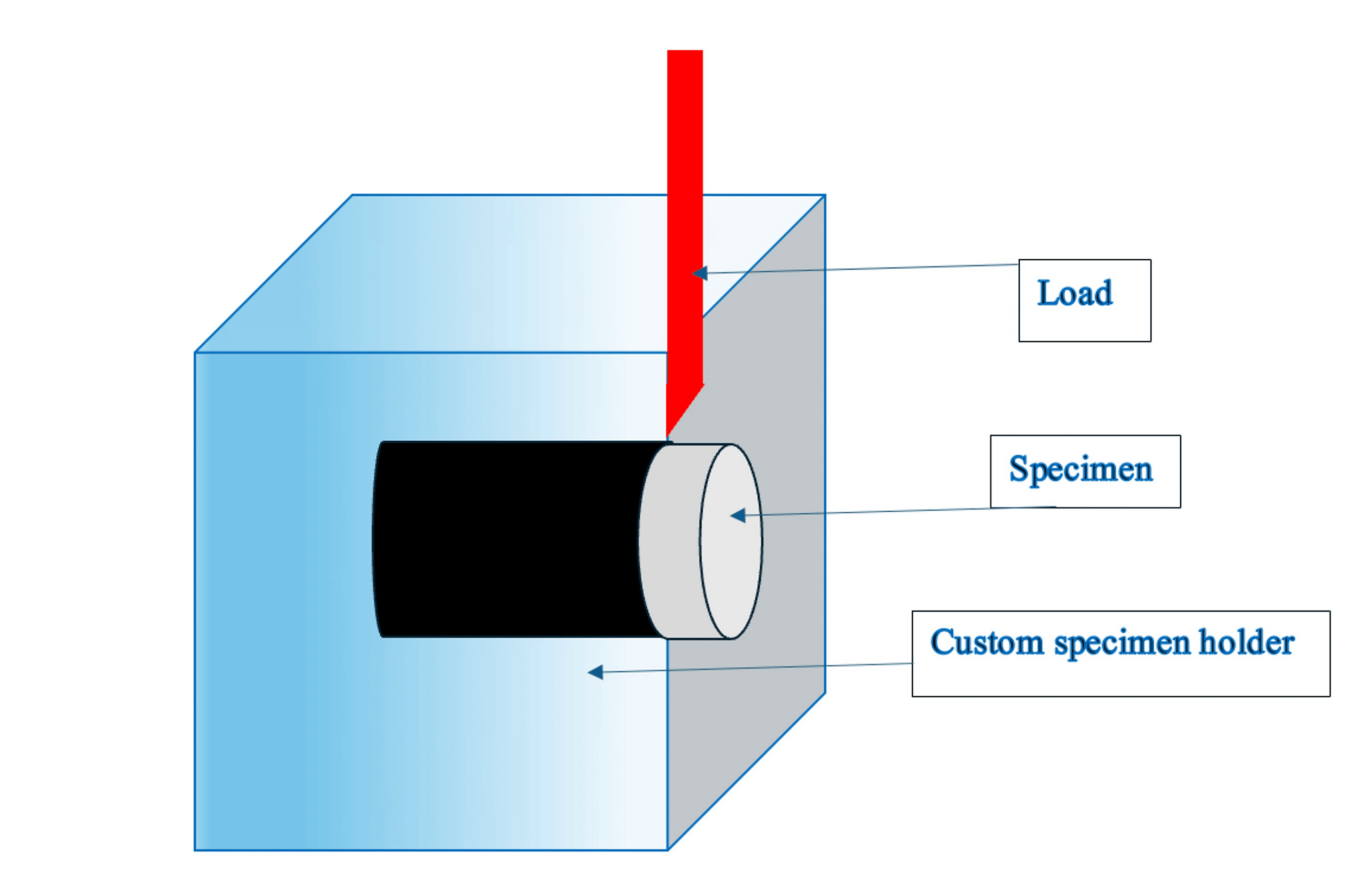
Diagrammatic representation of the shear bond strength test

Shear bond strength between the metal and ceramic was assessed using a Universal Testing Machine (3382 series; Instron, Norwood, MA, USA) equipped with a 0.5 mm thick bevel-shaped rod and a 5000 N load cell. Tests were conducted at a crosshead speed of 1 mm/min. Specimens were securely positioned in a custom-made apparatus (Figure 1) to ensure that shear forces were applied exclusively at the metal-ceramic interface (comprehensive data are included in Appendix A and Appendix B for reference).

The failure mode of each specimen was examined using a stereomicroscope (SZB-47A; PSAW) at 10× magnification. Surface microstructure and elemental composition were analyzed for one specimen from each group using scanning electron microscopy coupled with Energy Dispersive X-Ray Spectroscopy (EVO 18; ZEISS, Oberkochen, Germany). Specimens were mounted on metallic stubs using double-sided adhesive tape and coated with gold via a sputter coater to enhance conductivity. Imaging was performed at an accelerating voltage of 20 kV and a working distance of approximately 13.9 mm, at varying magnifications, using Smart scanning electron microscopy (SEM) software for imaging and EDAX APEX software for energy dispersive X-ray spectrometry (EDS) analysis. During EDS, a beam of accelerated electrons was focused on the specimen, stimulating the emission of characteristic X-ray peaks to identify the elemental composition.

The recorded shear bond strength values and failure modes were collected, tabulated, and statistically analyzed using IBM SPSS Statistics for Windows, Version 26.0 (Released 2018; IBM Corp., Armonk, NY, USA). A two-way ANOVA was performed to assess the effects of alloy type and surface treatment on metal-ceramic shear bond strength. Post hoc analyses were conducted using the Tukey test for comparisons among cast alloys and Bonferroni correction for comparisons between different treatments. Results were presented in graphs and tables. Statistical significance was set at p < 0.05.

## Results

The mean shear bond strength (MPa) among six groups with different alloy compositions and surface treatments (Table 1) was analyzed using IBM SPSS Statistics for Windows, Version 26.0. A two-way ANOVA was performed to evaluate the effects of alloy type and surface treatment on metal-ceramic shear bond strength. No significant interaction was observed between groups (different alloy concentrations) and treatment (oxidative heat treatment with or without air abrasion) on metal-ceramic shear bond strength (F(2, 102) = 0.0041; P = 0.96) (Table 2).

**Table 1:** Mean ± SD of shear bond strength for different groups of alloy versus surface treatment

Alloy preparation	Surface treatment	Number (n)	Mean (MPa)	SD (MPa)
100% fresh alloy (C0)	Oxidative heat treatment (S0)	18	62.4	9.4
100% fresh alloy (C0)	Oxidative heat treatment + air abrasion (S1)	18	66.9	15.1
Total for C0		36	64.65	12.6
50% fresh alloy + 50% remnant of first casting (C1)	Oxidative heat treatment (S0)	18	56.8	13.5
50% fresh alloy + 50% remnant of first casting (C1)	Oxidative heat treatment + air abrasion (S1)	18	61	15.1
Total for C1		36	58.8	14.3
50% fresh alloy + 50% remnant of second casting (C2)	Oxidative heat treatment (S0)	18	45	14.4
50% fresh alloy + 50% remnant of second casting (C2)	Oxidative heat treatment + air abrasion (S1)	18	47.7	15.2
Total for C2		36	46.3	14.7
Total	Oxidative heat treatment (S0)	54	54.7	14.4
	Oxidative heat treatment + air abrasion (S1)	54	58.5	16.9
	Total	108	56.6	15.7

**Table 2:** Comparison of different alloys and surface treatments regarding shear bond strength (two-way ANOVA test) ** indicates statistically significant difference using using two-way ANOVA.

Subgroups of the experiment	Type III sum of squares	df	Mean square	F	P-value
Groups for alloy	6297.6	2	3148.8	16.09	P = 0.001**
Surface treatment	390.3	1	390.3	1.99	P = 0.16
Groups for alloy × treatment	16.1	2	8.06	0.041	P = 0.96

However, a significant effect of fresh versus recast nickel-chromium alloys on metal-ceramic shear bond strength was found (F(2, 102) = 16.09; P = 0.001) (Table 2). In contrast, the type of surface treatment applied to the castings did not significantly affect metal-ceramic shear bond strength (F(2, 102) = 1.99; P = 0.16) (Table 2).

Pairwise comparison of shear bond strength between fresh and recast castings using the post hoc Tukey test revealed that the shear bond strength of C0 was similar to C1 (P = 0.25). Both C0 and C1 showed higher metal-ceramic shear bond strength compared to C2 (P = 0.001) (Table 3). Fracture modes were predominantly mixed (Table 4). 

**Table 3:** Pairwise comparison of shear bond strength for fresh and repeated casting (post hoc Tukey test) ** indicates statistically significant difference using the post hoc Tukey test.

Alloy group	Compared alloy group	Mean deviation	P-value
100% fresh alloy (C0)	50% fresh alloy + 50% remnant of first casting (C1)	5.76	P = 0.25
100% fresh alloy (C0)	50% fresh alloy + 50% remnant of second casting (C2)	18.2	P = 0.001**
50% fresh alloy + 50% remnant of first casting (C1)	50% fresh alloy + 50% remnant of second casting (C2)	12.5	P = 0.001**

**Table 4:** Failure mode

Alloy group	Surface treatment	Adhesive, n (%)	Cohesive, n (%)	Mixed, n (%)	Total, n (%)
100% fresh alloy (C0)	Oxidative heat treatment (S0)	1 (5.6)	0	17 (94.4)	18 (100)
100% fresh alloy (C0)	Oxidative heat treatment + air abrasion (S1)	0	0	18 (100)	18 (100)
50% fresh alloy + 50% remnant of first casting (C1)	Oxidative heat treatment (S0)	2 (11.1)	0	16 (88.9)	18 (100)
50% fresh alloy + 50% remnant of first casting (C1)	Oxidative heat treatment + air abrasion (S1)	1 (5.6)	0	17 (94.4)	18 (100)
50% fresh alloy + 50% remnant of second casting (C2)	Oxidative heat treatment (S0)	1 (5.6)	0	17 (94.4)	18 (100)
50% fresh alloy + 50% remnant of second casting (C2)	Oxidative heat treatment + air abrasion (S1)	1 (5.6)	0	17 (94.4)	18 (100)

Semiquantitative analysis using SEM-EDS indicated that chromium concentrations remained stable, whereas molybdenum levels decreased (Table 5). Nickel was least affected by recasting. Chromium content remained within the expected range, except for C2S1 (30.1%). Molybdenum generally decreased across successive recasting cycles, with an anomalously high value in C1S1 (21%). The molybdenum level in the heat treatment with air abrasion group was lower than that in the heat treatment-only group. Manganese was absent in the oxidative heat treatment group. Barium was detected only in trace amounts (0.2-0.4%).

**Table 5:** Semiquantitative analysis using SEM-EDS of chemical elements after fracture Al, aluminum; Ba, barium; C, carbon; Cr, chromium; EDS, energy dispersive X-ray spectrometry; Fe, iron; K, potassium; Mg, magnesium; Mn, manganese; Mo, molybdenum; Ni, nickel; O, oxygen; SEM, scanning electron microscopy; Si, silicon; Ti, titanium

Group	C (%)	O (%)	Al (%)	Si (%)	K (%)	Ti (%)	Cr (%)	Mn (%)	Fe (%)	Ni (%)	Mo (%)	Ba (%)	Mg (%)
C0S0	12.6	30.3	4	5.1	0.5	2.2	16.2	0	0	24.4	4.6	0.2	0
C0S1	11.8	27	2.3	5	0.8	2	16.4	0.4	0	29.4	0	0	5
C1S0	10.8	32.7	4.3	6.5	1.4	4.3	15.6	0	0	20.7	3.6	0	0
C1S1	30.9	2.1	0	2.8	0	0	11	0.7	0	31.5	21	0	0
C2S0	14.4	27.7	4	5.7	1	3	15.3	0	0	25.2	3.6	0.2	0
C2S1	7.3	20.2	1.9	6.3	0.8	2.3	30.1	0.2	0.1	28.6	1.9	0.4	0

## Discussion

Two-way ANOVA revealed no significant interaction between groups (different alloy concentrations) and treatment (oxidative heat treatment with or without air abrasion) on metal-ceramic shear bond strength (F(2, 102) = 0.0041; P = 0.96). However, pairwise comparison using the post hoc Tukey test showed that C0 and C1 had significantly higher metal-ceramic bond strength compared to C2 (P = 0.001). Therefore, the null hypothesis was partially rejected.

According to the International Organization for Standardization (ISO) 9693, the three-point bend test is the standardized method for evaluating metal-ceramic bond strength, specifying a minimum requirement of 25 MPa [25]. In the present study, the shear bond strength test was used because it supports specimens fully and applies stress directly at the metal-ceramic interface [26], minimizing the effect of the alloy’s Young’s modulus [18,27].

Incorporation of 50% fresh alloy into previously cast buttons or sprues is recommended for recasting metal-ceramic restorations [1,18,28]. A 1:1 mixture of fresh and first-generation cast alloy has been shown to yield metal-ceramic bond strengths exceeding the ISO threshold of 25 MPa, as confirmed in prior studies [8,27]. Reisbick and Brantley demonstrated that tensile strength remains unaffected after up to three recasting cycles, while other studies suggest that recasting may be viable for up to four generations based on evaluations of key physical properties [1].

In this study, shear bond strength decreased in subsequent castings compared to group C0. However, C0 and C1 exhibited nearly equivalent shear bond strengths and could be used safely without compromising mechanical properties. In contrast, both C0 and C1 showed significantly higher shear bond strength than C2, which contrasts with findings by James et al. [27] and Atluri et al. [29].

Following casting, the surface of dental alloys is typically contaminated with residual investment material and metal oxides [30]. Various surface treatment protocols have been proposed to improve metal-ceramic bonding, including oxidative heat treatment and air abrasion [10], both of which were employed in this study. Bond strength increased after air abrasion, likely due to the formation of an oxide layer. Sced and McLean [12] reported that air abrasion enhances mechanical interlocking by increasing the surface area available for bonding, consistent with the present findings, though the difference was not statistically significant. In contrast, Henriques et al. reported that air abrasion does not completely remove the oxide layer formed during oxidation heat treatment and may leave surface contaminants that alter the alloy’s chemical composition [16]. Once the original oxide layer is removed, it cannot be reformed with the same structural and chemical characteristics, resulting in a less effective bonding interface. While initial oxidative heat treatment of the first-cast metal was sufficient for optimal metal-ceramic bonding, repeated oxidation following subsequent recasting did not further enhance interfacial bond strength [30].

Fracture modes were analyzed using a stereomicroscope. Mixed failures were most common, followed by adhesive failures. Mixed failures occurred because the bond was strong enough to prevent complete adhesive separation, but variations in oxide layer thickness, elemental loss during recasting, and surface roughness created weak zones, distributing stress across both metal and ceramic and resulting in combined fracture modes [16,24]. No cohesive failures were observed, indicating strong shear bonding between the ceramic and base metal alloy. A weak correlation was found between failure modes and metal-ceramic bond strength. Henriques et al. [16] reported that oxidative heat treatment negatively affects metal-ceramic bonds, as preoxidized specimens exhibited adhesive failure, whereas non-preoxidized specimens displayed both adhesive and mixed failures.

SEM-EDS analysis was performed on one randomly selected specimen per group for qualitative assessment of elemental distribution, as all specimens were processed under comparable compositions and conditions. The results indicated that alloy type and surface elemental composition influenced bond strength upon recasting. Chromium, a major oxide-forming element, remained stable in fresh and first-generation recast groups but increased markedly in C2S1 (30.1%), which may affect castability. Although Cr₂O₃ contributes to ceramic bonding, excessively thick oxide layers may act passively and hinder metal-ceramic bonding [8]. Molybdenum, important for corrosion resistance and oxide layer quality, gradually decreased with each recasting cycle, notably in C2 groups, correlating with lower shear bond strength, except in C1S1, likely due to technical factors or localized inhomogeneity in oxide layer disruption during abrasion. Airborne particle abrasion increased surface roughness, exposing bulk nickel and improving mechanical retention, but also removed favorable oxides like Mo and Al, potentially reducing chemical bonding efficiency. The presence of barium in several samples likely originated from contamination from investment materials or furnace residues.

Clinical implications

When used appropriately, a single recasting cycle of Ni-Cr base metal alloys with 50% fresh alloy provides a cost-effective option for fabricating metal-ceramic prostheses without compromising bond strength. This approach aligns with the principles of green dentistry by reducing material waste and promoting sustainability.

Limitations

Limitations of this study include its controlled laboratory setting and the absence of aging protocols such as artificial saliva storage, thermocycling, or cyclic loading, which would more closely simulate clinical conditions. In addition, detailed evaluation of alloy composition and structural homogeneity in relation to phases and grain size could provide further insight. While the shear bond strength test allows direct stress application at the metal-ceramic interface and minimizes the influence of the alloy’s elastic modulus, it limits comparability with ISO 9693-based studies and restricts the generalizability of the findings.

## Conclusions

Based on the findings of this study, the addition of up to 50% remnants of cast alloy was considered acceptable, as there was no significant difference in shear bond strength between groups C0 and C1. However, group C2 exhibited significantly lower shear bond strength, indicating pronounced deterioration after two casting cycles. Surface treatments were found to enhance shear bond strength, but they could not fully restore the bond strength lost due to repeated alloy reuse. In addition, changes in the elemental composition of the alloy surface with multiple castings also influenced the metal-ceramic bond strength.

## Figures and Tables

**Figure 2:**
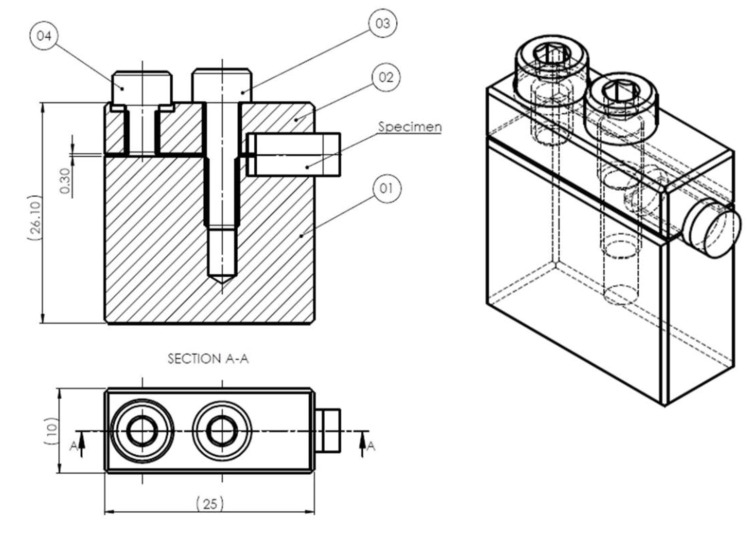
Diagrammatic representation of custom specimen holder Part no. 1 – Holder plate bottom (25 × 20 × 10 mm), material: SS-304 (stainless steel); Part no. 2 – Holder plate top (25 × 20 × 6 mm), material: SS-304 (stainless steel); Part no. 3 – Allen screw (M4 × 16 mm), material: standard stainless steel; Part no. 4 – Allen screw (M4 × 16 mm), material: standard stainless steel

**Figure 3:**
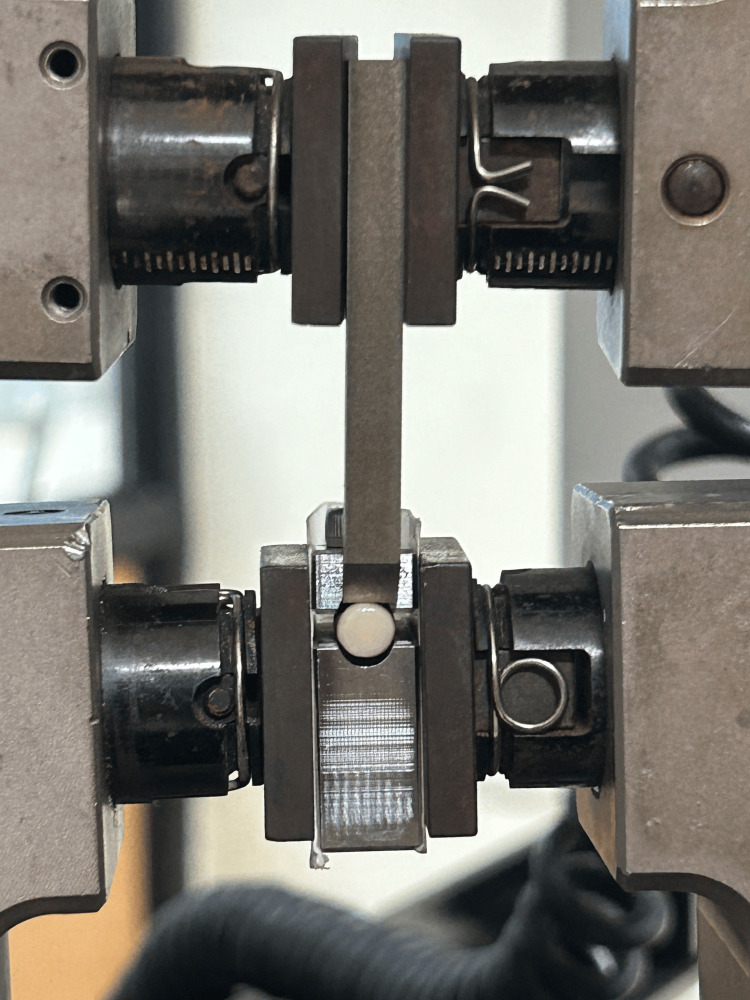
Specimen mounted on universal testing machine using custom-made apparatus for testing

## Appendices

Appendix A

A diagrammatic representation of the custom specimen holder is shown in Figure 2.

Appendix B

A custom specimen holder assembly was fabricated using a CNC milling machine (Model Winner) to securely position the specimens and facilitate the application of load along the projected diameter, enabling evaluation of the bond strength between the two materials.

The specimen holder assembly was operated by first loosening Allen screw no. 3, maintaining a small gap of approximately 0.3-0.5 mm. The specimens were then placed in the half-round slot of the holder, ensuring that proper gripping was clearly visible. Once the specimens were correctly positioned, Allen screw no. 3 was gently tightened to hold them firmly in place without causing damage, and the holder with the specimen was carefully placed into the testing machine (Figure 3) to ensure proper alignment with the test setup.
